# Vital Statistics*Read at Corpus Christi meeting of the Association of Texas Health Officers.

**Published:** 1908-07

**Authors:** S. Burg

**Affiliations:** City Health Officer, San Antonio, Texas


### Vital Statistics.*

BY DR. S. BURG, CITY HEALTH OFFICER, SAN ANTONIO, TEXAS.

The great chemist Liebig once said that the degree of civilization of a country is measured by the amount of soap it uses. If I should be permitted the use of a similar phrase I might say, that the degree of civilization of a country is measured by the way its vital statistics are registered and by the degree of its perfection. The necessity of good records of vital statistics is obvious to every physician, and also to the educated layman, still our country stands in this respect far behind other civilized countries. The proper knowledge of the fluctuations in human life in regard to morbidity and mortality is so essential for the knowledge of the causes of diseases, especially those of an epidemic character, that it is hard to understand why State, county and city authorities are so reluctant in adopting and enforcing stringent measures of registration of

*Read at Corpus Christi meeting of the Association of Texas Health Officers.

vital statistics. If they are adopted, they are either badly carried out or entirely ignored. We must admit, though, that our last Legislature has enacted measures which can be considered a step forward in the right direction, and the State health authorities are doing their best to bring about satisfactory conditions, but so far the results are far from being satisfactory.

It affords me, therefore, a feeling of pride to state that San Antonio is about the only city in Texas that can aspire to the claim of having satisfactory records of vital statistics. The first requirement is ordinances enacted by the city council making it obligatory on the practicing physicians to report infectious and contagious diseases and also every case of death by filling out a death certificate, giving all the necessary data in connection with the death. Another ordinance requiring physicians and midwives to report births is indispensable. If you add to this a systematized health office, with the proper clerical help under the supervision of a board of health, you have the necessary means of keeping proper records of vital statistics. In providing for all these requirements San Antonio has been leading. It is true that the costs were considerable, the amount allotted in the coming year's budget for it being $23,000, but hardly as such an amount of money been appropriated for a better purpose. There is a permanent record kept in a well-bound book of every death occurring in the city, giving all the necessary data as to name, age, cause of death, color, sex, nativity, attending physician, place where buried, place where disease was contracted, how long a citizen of the city. No burial permit is issued unless a death certificate is furnished by a legally practicing physician, or a coroner if no medical attendant was employed.

Another record is kept of all contagious diseases, which the ordinance requires physicians to report to the health office. Among the reportable diseases, the ordinance mentions the following: Smallpox, diphtheria, scarlet fever, measles, typhoid fever, typhus, yellow fever, cholera, bubonic plague, severe forms of malarial fever, and other severe infectious or contagious diseases. Whenever a report of an infectious or contagious disease is made, notice is sent to the school board and public library of such a disease existing in such and such a place, so that the necessary measures can be taken to prevent the spread of the disease. Fumigation also takes places of the house after the disease is ended by either recovery or death. A record is kept giving data as to sex, race, color, parents, and their nativity, date and place of birth, whether alive or stillborn. In order to facilitate such record, postals are sent to every practicing physician containing all the questions regarding the different data for contagious diseases and for birth, which are filled out and mailed to the health office. Death certificates are furnished and brought to the health office by either the undertaker or by the respective parties, to get the burial permit. Question blanks for eliciting the origin of infectious diseases and blanks giving information of how to prevent infectious diseases are sent to the houses from where a contagious disease has been reported. In such a way the health office is enabled to keep records of vital statistics and to work out reports and tables of information which can only benefit our community and be instructive to others. Let us hope that all other communities in Texas will follow suit and help Texas to stand foremost on the question of vital statistics.

